# An atypical presentation of hypothyroidism with extremely exaggerated functional impairment

**DOI:** 10.1002/ccr3.7708

**Published:** 2023-07-18

**Authors:** Alireza Arezoumand, Sahar Nazari, Kimia Jazi, Mohammad Bagherzade, Mohammad Mehdi Riahi, Melika AkbariMehr, Narges Kanganee, Maryam Masoumi

**Affiliations:** ^1^ Student Research Committee, Faculty of Medicine Medical University of Qom Qom Iran; ^2^ Clinical Research and Development Center, Shahid Beheshti Hospital Qom University of Medical Sciences Qom Iran; ^3^ Neuroscience Research Center, Faculty of Medicine Qom University of Medical Sciences Qom Iran

**Keywords:** hypothyroidism, myopathy, myopathy‐associated hypothyroidism, pseudo‐polymyositis

## Abstract

**Key Clinical Message:**

Myopathy‐related symptoms are rare manifestations of hypothyroidism. Clinicians should consider hypothyroid myopathy as one of the possible diagnoses for patients with proximal weaknesses.

**Abstract:**

Myopathy‐related symptoms are rare manifestations of hypothyroidism. Clinicians should consider hypothyroid myopathy as one of the possible diagnoses for patients with proximal weaknesses. We report a 34‐year‐old woman, presenting with a new atypical musculoskeletal manifestation of hypothyroidism mimicking polymyositis.

## 1 INTRODUCTION

Pathological thyroid hormone deficiency known as hypothyroidism is commonly found in general population. More frequently in women and elderly, a European meta‐analysis reported that the prevalence of overt and clinical hypothyroidism considers 0.37% and 3.8% as well as 226/100,000 predicted incidence.^1^ Symptoms are mostly nonspecific including constipation, fatigue, and cold nonintolerance, thus, the detection of high thyroid‐stimulating hormone (TSH) and low free thyroxin (fT_4_) concentrations is the key diagnostic approach.^2^ Less common symptoms include dry skin, myxedema, hoarseness, normochromic, and normocytic but infrequently macrocytic anemia, increased thrombosis risk, as well as neurological (encephalopathy and carpal tunnel syndrome). Musculoskeletal manifestations present in 25%–79% of adults suffering hypothyroidism such as myalgia, increased serum creatine kinase (CPK) levels, stiffness, and cramps.^1^

Herein, we report the first case of a 34‐year‐old woman, presenting with polymyositis that ended with a new atypical musculoskeletal manifestation of hypothyroidism.

## 2 CASE PRESENTATION

A 34‐year‐old woman without any past medical history presented to emergency room with intensified generalized weakness over the past 2 months. She could not walk without the help of her husband, which was aggravated in the past 2 months. She complained of an insidious proximal weakness in both upper and lower limbs 2 months ago. Her hips, thighs, shoulders, and neck were all involved with progressively worsening pain to the extent of being hardly able to hair combing, picking up her child, climbing the stairs, and even walk. She also complained of constipation and generalized abdominal pain over past 2 months. In physical examination, evaluating the force of limbs out of 5, the upper limb in proximal part got 3 and the distal part scored 5. The lower limb proximal and distal parts ranked 3 and 5, respectively. Moreover, the force of cervical flexor muscle reached 4. Before the crisis, she stated that she had no functional impairment. Her past medical history was negative, and she declined any family history of rheumatologic disorders or the same symptoms.

Laboratory results of the first day of admission were as follows (Table 1): CPK (creatinine phosphokinase): 5120 U/L, LDH (lactate dehydrogenase): 934 U/L (225–500), aldolase: 14.5 U/L (<7.6), Urea: 45 mg/dL (10–50), creatinine: 1.5 mg/dL (0.6–1.2), CRP (c‐reactive protein) <3 mg/dL (negative: <6, Equivocal: 6–9, positive: >9), ESR (erythrocyte sedimentation rate) 1 h: 13 mm/h. (<20), TSH (thyroid‐stimulating hormone) >100 mIU/L (0.27–4.2), total T4 (thyroxine) <0.5 μg/dL (4.5–12.6), anti‐TPO (anti‐thyroid peroxidase antibody): 906 IU/mL (<5.61), COVID‐19 RT‐PCR: Negative. Ultrasonography indicated Fatty liver grade 1 and the other parts of abdomen and pelvis were normal.

**TABLE 1:** Serial laboratory results.

Test, unit	Result							Reference range
Blood biochemistry								
	Admission	First day	Second day	Third day	Fourth day	Fifth day	Discharge	
BS, mg/dL	106							70–120
AST, U/L	161							Up to 35
ALT, U/L	84							Up to 45
ALP, U/L	133							98–279
LDH, U/L	934	907	969	861	757	671	741	225–500
CRP, mg/dL	<3							Negative: <6, Equivocal: 6–9, Positive: >9
Creatinine, mg/dL	1.5	1.6	1.5		1.5	1.4	1.3	0.6–1.2
Urea, mg/dL	45		29	22	30	32	34	10–50
Uric Acid, mg/dL	3.9							Male 3.4–7 Female 2.4–5.7
Na, mmol/L	133	143	138		139	138	138	135–148
K, mmol/L	3.8	4.1	4		4.5	3.9	4.3	3.5–5.3
Ca, mg/dL	10.3							8.8–10.2
P, mg/dL	3.6							2.5–5
Mg, mmol/l	2.4							1.9–2.5
Alb, g/dL	4.7							3.6–4.8
CPK Total, U/L	5120	4059	3056	2668	2275	1866	1718	24–170
Aldolase, U/L	14.5							<7.6
CK‐MB, IU/L	62							<24
Troponin I ultra, ng/mL	2.00							Without myocardial damage: <19
Hematology								
WBC, μL	9400							4000–11,000
RBC, 10^6/μL	3.88							4.2–6.3
Hb, g/dL	12.2							12–16
Hematocrit, %	36.1							30–45
MCV, fL	93.0							80–100
MCH, pg	31.4							27–32
MCHC, g/dL	33.7							33–38
Platelet, μL	290,000							150,000–450,000
Neutrophil, %	60							—
Lymphocyte, %	36							—
Serology and Endocrinology								
ESR 1 h, mm/h.	13							20>
COVID‐19 RT‐PCR	negative							
Urine 24 h./Pr, mg/24 h.			35					24–141
Urine 24 h./ Urea, g/24 h.			14					13–36
Urine 24 h./Cr, mg/24 h.			1140					600–1800
Urine 24 h./Volume, mL			2000					—
TSH, μIU/L	>100							0.27–4.2
T4, μg/dL	<0.05							
Anti‐TPO AB, IU/mL	906.03							<5.61
Anti‐ds DNA AB, IU/mL		10						Negative: <100, Positive: > = 100
C3, mg/dL		108						90–180
C4, mg/dL		33						10–40
CH50, U/mL		94						> = 90
FANA		Negative						Negative: no reaction at 1:100 Trace: positive reaction at 1:100 Positive: positive reaction at 1:320 or more

Abbreviations: Alb, albumin; ALP, alkaline phosphatase; ALT, alanine transaminase; Anti‐ds DNA AB, anti‐double stranded DNA antibody; anti‐TPO AB, anti‐thyroid peroxidase antibody; AST, aspartate aminotransferase; BS, blood sugar; C3 and C4, complement 3 and 4; Ca, calcium; CH50, a total hemolytic complement; CK‐MB, creatine phosphokinase‐myocardial band; CPK, creatine phosphokinase; CRP, c‐reactive protein; ESR, erythrocyte sedimentation rate; FANA, fluorescent antinuclear antibody; Hb, hemoglobin; K, potassium; LDH, lactate dehydrogenase; MCH, mean corpuscular hemoglobin; MCHC, mean corpuscular hemoglobin concentration; MCV, mean corpuscular volume; Mg, magnesium; Na, sodium; P, phosphorus; RBC, red blood cell; T4, thyroxine; TSH, thyroid‐stimulating hormone; WBC, white blood cell.

High levels of CPK, aldolase, and LDH at first presentation (Table 1), as well as her first clinical presentation, led to a possible diagnosis of inflammatory myopathies such as polymyositis, and immune‐mediated necrotizing myopathy. We ordered pulse methylprednisolone therapy to avoid further inflammatory complications before the results of preclinical assessment get ready, however, hypothyroidism myopathy according to her TSH levels kept us waiting for the results of further evaluations. Consequently, we started oral levothyroxine 1.6mcg/kg/day as the patients weighted 80 kgs, which is approximately equivalent to 150 microgram/day. Although intravenous (IV) levothyroxine is the choice in these conditions with severe manifestations, we had to start oral therapy since IV levothyroxine was not available.

To further evaluate inflammatory myopathies, EMG‐NCS (electromyography‐nerve conduction velocity) and more laboratory tests were conducted: creatinine: 1.3 mg/dL, protein in 24 h urine: 35 mg/24 h., urea in 24 h urine: 14 g/24 h., Cr: 1140 mg/24 h., 24 h urine volume: 2000 mL, anti‐ds‐DNA (Anti‐double stranded DNA) <10 IU/mL (negative<100, positive> = 100), C3: 108 mg/dL (90–180), C4: 33 mg/dL (10–40), CH50: 94 U/mL (> = 90), FANA (fluorescent antinuclear antibody): negative (no reaction at 1:100). The first EMG‐NCV indicated normal sensory/motor NCS (nerve conduction studies) and needle exam indicated no myopathy pattern and no spontaneous discharge. To verify the results of the first EMG‐NCV, the second was conducted during the first follow‐up after 2 weeks showing normal outcomes as well.

**FIGURE 1:**
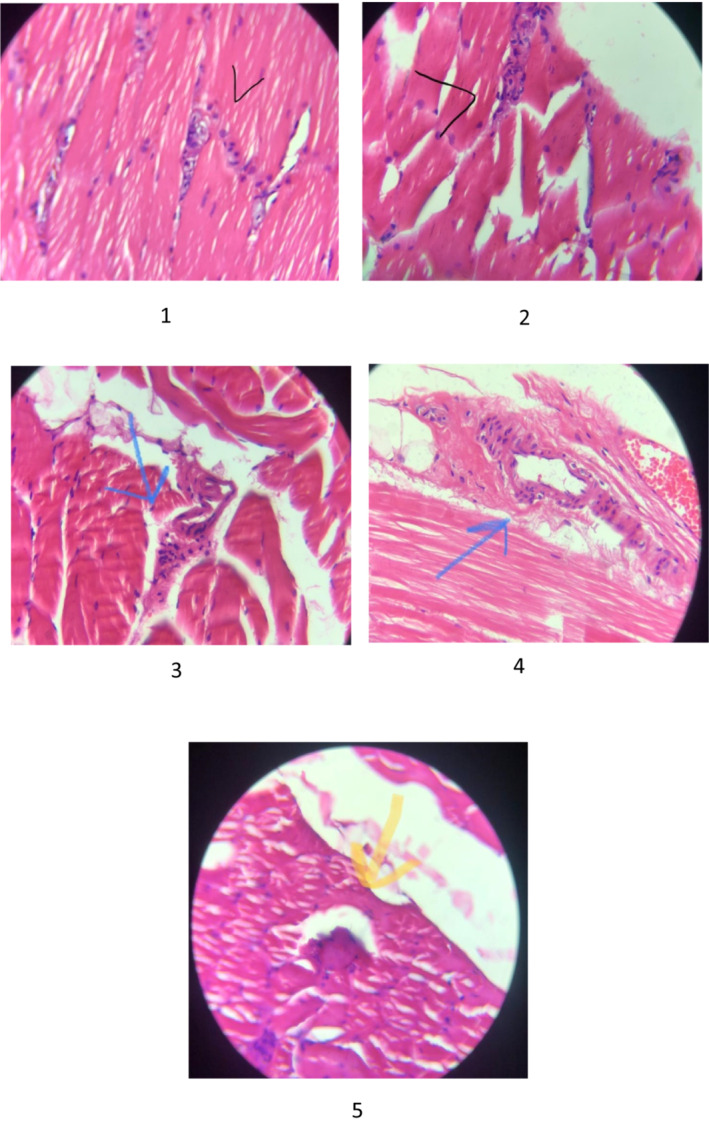
Microscopic findings of biopsy of quadriceps skeletal muscle; (1, 2): mild variation in size of fiber, nuclear sarcolemmal proliferation, (3, 4): few lymphocytic infiltrations around small arteries, (5): basophilic change in a few fibers' arteries.

Microscopic findings of biopsy of quadriceps skeletal muscle were as follows (Figure 1): skeletal muscle with mild variation in size of myofibers, few nuclear centralizations and mild increased sarcolemmal nuclei, also a few degenerated and pale myofiber with a few lymphocytic inflammations around small vessels. According to pathological reports, diagnosis was nonspecific myopathy, thus we did not start acute polymyositis treatment. Continuing the observations, during her admission, CPK, LDH, and creatinine concentrations retained higher than reference range (Table 1), however, gradual decrease was found after starting thyroid hormone therapy.

Considering microscopic findings, this case was suggestive for myopathy of hypothyroidism, and the patient was discharged prescribed with levothyroxine 150 microgram/day. Her follow‐up in 1 month had TSH = 14.06 μIU/L (0.4–4.2) and after 2 months, her laboratory test indicated TSH = 0.7 μIU/L (0.35–6) and free T4 = 1.6 μg/dL (0.7–2.0), and she had normal muscle enzymes and physical examination, and all the symptoms disappeared along with TSH decrease.

## 3 DISCUSSION

Muscle involvement is a prevalent problem in both congenital and adult hypothyroidism, which can vary intensively.^3^ Regardless of the fact that these were rarely the initial symptoms of the disease, in a limited sample, 79% of patients with recently diagnosed hypothyroidism experienced of weakness, fatigue, cramps, and myalgias.^4^ Our case, although similar to previous reports in generalized weakness and progressive myalgia, she was the first case presenting with polymyositis typical signs and symptoms caused by hypothyroidism. Nonspecific muscle stiffness or diffuse muscle pain are frequent symptoms of hypothyroidism, which may be in correlation with an increase in the levels of muscle enzymes^5^; however, to date, there is no report of inability to walk or complete functional impairment. In line with our patient's symptoms, proximal muscle weakness gradually increases in time. Hypothyroid myopathy can occasionally be more severe and accompanied by a noticeable rise in muscle enzymes.^6^, ^7^ Consistently, the serum CPK level raised in 57 to 90% of individuals with hypothyroid myopathy, long before the onset of typical clinical symptoms of hypothyroidism. Although the level of CPK and the level of TSH have been indicated to correlate in some cases, the level of CPK is not directly related to the severity of clinical symptoms.^8^

The exact mechanism of hypothyroid‐associated‐myopathy remains uncertain. T4 deficiency causes disturbances in glycogenolysis and mitochondrial oxidative metabolism. Consequently, resulting in muscular dysfunction.^9^ One of the manifestations is specific atrophy of type 2 fibers, which are more reliant on glycolysis for their energy source. Rhabdomyolysis of muscle cells can happen with severe or persistent oxidative damage.^10^ Several cases of extremely severe rhabdomyolysis associated with hypothyroidism have been documented.^11^, ^12^

A hypothyroid evaluation is necessary if any of the aforementioned symptoms or signs cannot be explained by other identified causes. EMG could be helpful in order to distinguish hypothyroid myopathy from inflammatory myositis or other neuromuscular disorders. Nearly 50% of hypothyroid individuals with proximal muscle weakness have normal EMGs, and the rest may show myopathic alterations with an increased low amplitude, polyphasic potentials, and rarely increased insertional activity.^13^ Muscle biopsy is usually normal or may contain a few nonspecific changes such as Mild, focal necrosis, degeneration of muscle fibers and mild inflammatory infiltrates. Indeed, extensive inflammatory changes can suggest an alternative diagnosis such as polymyositis.^14^

Thyroid hormone replacement therapy is efficient in treating hypothyroid myopathy. Within a few weeks, the serum CPK level would return to normal range after a rapid decline, even earlier than TSH.^15^ Notably, clinical symptoms considering muscular weakness, recover more slowly.^5^ In this report, normal physical examinations, muscle enzymes, and disappearance of symptoms after 2 weeks of levothyroxine therapy also confirmed hypothyroid myopathy. Thus, considering hypothyroidism as one of possible differential diagnosis in patients presenting with myopathy symptoms even very severe such as impaired gait should be considered along with inflammatory or neurological diseases.

## 4 CONCLUSION

Hypothyroidism with the first presentation mimicking inflammatory myopathies, called hypothyroidism myopathy, is very rare. Currently, we reported the first case of gait and functional impairment, showing progressive pattern in 2 months. Practitioners should precisely consider hypothyroidism as one of the first differential diagnosis while facing high muscle enzymes and recent proximal weakness in order to avoid further unnecessary evaluations and cost in the clinic.

## AUTHOR CONTRIBUTIONS

**Alireza Arezoumand:** Conceptualization; investigation; supervision; writing – original draft. **Sahar Nazari:** Data curation; writing – original draft. **Kimia Jazi:** Conceptualization; validation; writing – original draft. **Mohammad Bagherzade:** Project administration; validation. **Mohammad Mehdi Riahi:** Conceptualization; resources; supervision; writing – original draft. **Melika Akbarimehr:** Data curation; project administration; validation. **Narges Kanganee:** Conceptualization; resources. **Maryam Masoumi:** Conceptualization; supervision; visualization; writing – review and editing.

## FUNDING INFORMATION

This research was not funded.

## CONFLICT OF INTEREST STATEMENT

The authors declare no conflict of interest.

## CONSENT

Written informed consent from the patient's husband and herself has been obtained to publish this report in accordance with the journal's patient consent policy.

## Data Availability

The data that support the findings of this study are available on request from the corresponding author. The data are not publicly available due to their containing information that could compromise the privacy of research participants.
